# Strategies for Cancer Treatment Based on Photonic Nanomedicine

**DOI:** 10.3390/ma14061435

**Published:** 2021-03-16

**Authors:** Sueli Aparecida de Oliveira, Roger Borges, Derval dos Santos Rosa, Ana Carolina Santos de Souza, Amedea B. Seabra, Francesco Baino, Juliana Marchi

**Affiliations:** 1 Centro de Engenharia, Modelagem e Ciências Sociais Aplicadas, Universidade Federal do ABC, Avenida dos Estados, 5001 Santa Terezinha, Santo André 09210580, Brazil; sueli.oliveira@ufabc.edu.br (S.A.d.O.); derval.rosa@ufabc.edu.br (D.d.S.R.); 2 Centro de Ciências Naturais e Humanas, Universidade Federal do ABC, Avenida dos Estados, 5001 Santa Terezinha, Santo André 09210580, Brazil; roger.borges@aluno.ufabc.edu.br (R.B.); ana.galvao@ufabc.edu.br (A.C.S.d.S.); amedea.seabra@ufabc.edu.br (A.B.S.); 3 Department of Applied Science and Technology, Institute of Materials Physics and Engineering, Politecnico di Torino, Corso Duca degli Abruzzi, 10129 Torino, Italy; francesco.baino@polito.it

**Keywords:** nanomedicine, photonic, cancer treatment, photothermal therapy, photodynamic therapy, drug delivery systems

## Abstract

Traditional cancer treatments, such as surgery, radiotherapy, and chemotherapy, are still the most effective clinical practice options. However, these treatments may display moderate to severe side effects caused by their low temporal or spatial resolution. In this sense, photonic nanomedicine therapies have been arising as an alternative to traditional cancer treatments since they display more control of temporal and spatial resolution, thereby yielding fewer side effects. In this work, we reviewed the challenge of current cancer treatments, using the PubMed and Web of Science database, focusing on the advances of three prominent therapies approached by photonic nanomedicine: (i) photothermal therapy; (ii) photodynamic therapy; (iii) photoresponsive drug delivery systems. These photonic nanomedicines act on the cancer cells through different mechanisms, such as hyperthermic effect and delivery of chemotherapeutics and species that cause oxidative stress. Furthermore, we covered the recent advances in materials science applied in photonic nanomedicine, highlighting the main classes of materials used in each therapy, their applications in the context of cancer treatment, as well as their advantages, limitations, and future perspectives. Finally, although some photonic nanomedicines are undergoing clinical trials, their effectiveness in cancer treatment have already been highlighted by pre-clinical studies.

## 1. Introduction

### 1.1. The Challenge of Cancer Treatment

Cancer comprises a large group of diseases characterized by the appearance of structurally and functionally altered cells that proliferate uncontrollably, invade neighboring tissues, and lead to malignant tumors called neoplasms [1]. Cancer affects most organs and tissues, and the most common human tumors, the carcinomas, are responsible for more than 80% of cancer-related deaths, including those associated with lung, colorectal, stomach, liver, and breast, which are the most common causes of cancer deaths worldwide [2]. Despite the growing knowledge about hallmarks of tumor cells and their functioning in recent decades, cancer remains a challenging disease representing the second leading cause of death globally after cardiovascular pathologies. According to the World Health Organization (WHO), cancer was responsible for an estimated 9.6 million deaths, or one in six deaths, in 2018 worldwide [2].

One of the outstanding issues in the treatment of cancer is the heterogeneity of the tumor itself. Even a single person’s tumor contains diverse cells with different molecular characteristics and, consequently, different treatment sensitivity levels [3]. Cancer cells are characterized by genetic instability that is the source of the continuous appearance of different molecularly and functionally inside the tumor cell, making cancer a dynamic disease. That is why the most effective treatments of the disease must consider each patient’s specific molecular characteristics before and during the treatment; in other words, the treatments must be as personalized as possible, which converges with the current personalized medicine trends [4].

Genetic instability and heterogeneity are also associated with the appearance of resistance mechanisms in cancer cells to every current therapy, one of the significant causes of cancer treatment failure [5]. Thus, cancer therapies capable of curing remain a significant challenge, mainly in cancers that display cells with the multidrug resistance (MDR) phenotype. Cells presenting the MDR phenotype are characterized by cross-resistance to a wide array of anti-cancer drugs, harboring distinct structures and action mechanisms.

One of the main obstacles to curing cancer is metastasis, the most life-threatening event in cancer cases and one of the major causes of cancer-related fatalities worldwide [6]. Metastasis is a complex and multistep process during which cancer cells circulate through the body and invade different organs forming secondary tumors, also known as macrometastasis [7]. Metastatic cells are aggressive and resistant, and there is currently no effective therapy for the treatment of cancer at this stage which is responsible for 90% of deaths caused by cancer [8].

Surgery, chemotherapy, and radiotherapy, which were developed before 1975, are still the mainstay of cancer treatment. Surgeries are invasive procedures that require specific infrastructure, specialized professionals, and expenses with the procedure and maintenance of patients in a hospital, which can also deal with postoperative pains. Besides, surgeries are usually not effective in treating metastatic tumors, and, even in localized tumors, they may not be successful in clearing cancer within surgical margins, leading the patient to undergo a re-operation. Radiotherapy is generally well tolerated by most patients, although patients can experience side effects in some cases such as fatigue, tissue injury, inflammation, swelling, edema, and pain [9]. Unfortunately, although radiotherapy can reduce or eliminate tumors leading to remissions, the mutagenic actions of X-rays can induce the appearance of new second-site tumors years after the end of the therapy. While surgery and radiotherapy are the primary treatments used for local and non-metastatic cancers, anti-cancer drugs, such as chemotherapy, hormone, and biological therapies, have been the treatment of choice for metastatic diseases. Chemotherapy is based on administering anti-cancer drugs that can disrupt the cell cycle and induce cell death [10]. It is estimated that the proportion of patients that benefit from chemotherapy may be as low as 20% in specific tumor types. Indeed, a significant number of patients experience non-substantial tumor response after chemotherapy, yielding side effects like fatigue, cardiovascular and neurocognitive diseases, loss of fertility, and development of second cancers [11]. The difference between doses of chemotherapeutic agents able to elicit anti-tumor effects and those giving life-threatening toxicity is minimal, and the concomitant use of three or more agents frequently leads to high systemic toxicity, including peripheral neuropathy, bone marrow suppression, and kidney, liver, or cardiac toxicity [12,13]. Most of these cytotoxic drugs used in the treatments target processes in proliferating cells and do not distinguish healthy from cancer cells. Frequently, the most affected cells are those with high proliferative rates, such as hair follicles, the bone marrow and gastrointestinal tract epithelium, which explain the common side effects observed in patients during treatment [10]. Thus, it is practically impossible for cancer chemotherapy to achieve tumor remission without risk for potentially life-threatening adverse effects [14].

The lack of specific action upon cancer cells, high toxicity, development of MDR, low effectiveness upon metastatic cells, among other disadvantages showed by the traditional cancer therapies, have led to the development of many new treatment strategies and therapies known as targeted therapies. These new treatments act by blocking specific biological transduction pathways and/or cancer proteins involved in tumor growth and progression present in normal cells but are found overexpressed or mutated in cancer cells [10]. Unfortunately, most of these new treatments rarely achieve the effectiveness of traditional anticancer therapies in extending cancer patients’ survival and, like such approaches, they also fail to eradicate most types of tumors and metastasis, showing no curative outcome [15]. Thus, it is possible to conclude that the future of cancer treatment should be based on new anti-tumor drugs and new models of therapies capable of providing a better quality of life. In this scenario, photonic nanomedicine is highlighted as a therapeutic option to improve cancer treatment. Photonic nanomedicine can also combine two factors essential to any cancer treatment: temporal and spatial control of effects, which will be discussed further in the next section.

### 1.2. The Advantages of Photonic Nanomedicine for Cancer Treatment

Aiming to overcome limitations of traditional treatments, nanomedicine, i.e., medicine mediated by nanoparticles/nanostructured materials, has arisen as a promising therapy. Over the past few decades, there has been remarkable progress in developing and applying nanoparticles for more effective cancer treatment. The combination of therapeutic agents with nanoparticles aims to design structures with ideal sizes, shapes, and surface properties to increase their solubility, prolong their half-life, improve biodistribution, and reduce immunogenicity. Nanoparticles promote an improved permeability and retention effect, with spatial variations depending on different pH values of intracellular compartments, such as the endoplasmic reticulum (ER), the endocytic recycling compartment (ERC), the microtubule-organizing center (MTOC), and the multivesicular bodies (MVB) [15,16,17].

The size (10–200 nm) and surface chemistry of nanoparticle (or macromolecule) agents are crucial to promoting selective accumulation in tumors due to the impaired lymphatic drainage system. Shape, electrical charge, hydrophilicity, and circulation time in blood, are also responsible for the enhanced permeability and retention (EPR) effect displayed by nanoparticles. Since tumors have a high rate of nutrient consumption, promoting rapid growth of cancer cells, the EPR effect guarantees that most of the nanoparticles are accumulated in the cancer site. Also, imperfect blood vessels are generated during tumor growth, enabling the nanoparticles to penetrate into the tumor environment. In contrast, healthy tissue with regular blood vessel caliber acts as a barrier for circulating nanoparticles [17,18].

Photonic nanomedicine refers to a specific niche of nanomedicine, which combines photonics principles with nanomedicine [19]. In this sense, an intended therapeutic effect displayed by a nanoparticle is only triggered if a photon beam is applied, which makes it a stimulus-responsive therapy [20]. The interaction of photons with matter is due to its electromagnetic nature. Photons are elementary particles, consisting of the quantum of an electromagnetic field and the force carrier for the electromagnetic force [21]. Because of its quantic properties, photons can interact with electrons present in molecular orbitals of the matter, yielding chemical reactions, such as bond cleavage, physical phenomena like electron excitation, and photonic energy conversion into heat or light. The products from physical–chemical interactions between photons and matter can bring therapeutic effects in living tissues, as shown in the next sections. Also, because nanoparticles have a higher density of electrons on their surface as a consequence of their higher effective surface area, or because nanoparticles have the same size of the photon wavelength, some physical-chemical interactions can be further enhanced, making nanoparticles even more interesting platforms in biomedical applications [22,23].

Photonic nanomedicine can combine two factors that are essential to any cancer treatment: temporal and spatial resolution. The temporal resolution refers to the time needed in which therapy should act on the cancer site to bring therapeutic effect. Sometimes, therapy should be repeatedly applied, while sometimes only one dose is required, but in both cases, the therapy applications are managed to trigger as few side effects as possible [24]. The spatial resolution refers to the therapeutic need to act only in the cancer site, without affecting healthy cells or yielding systemic side effects [20]. While nanoparticles can accumulate or be locally applied into the cancer site, which means high spatial resolution, photonics as an external stimulus allow triggering the therapy at specific times under photon stimulation, which means high temporal resolution. Therefore, the combination of nanoparticles and photonics enables high spatial and temporal resolution, yielding fewer side effects and better patients’ quality of life.

**Figure 1:**
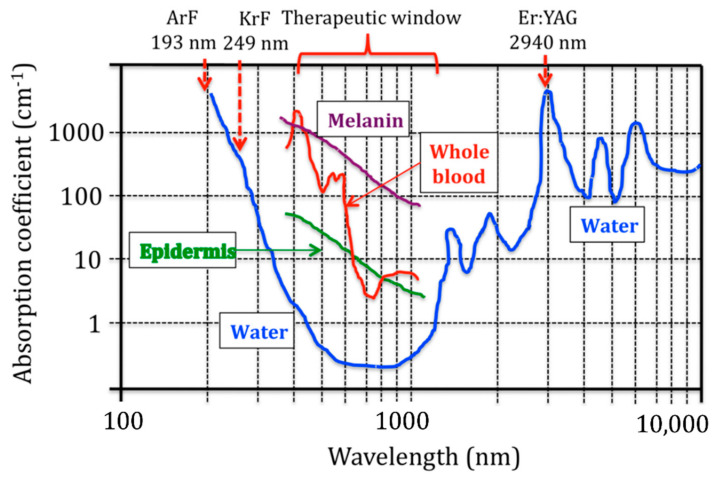
Absorption coefficient in the function of the light wavelength of the main components of several significant tissues: epidermis, water, whole blood, melanin. The therapeutic window refers to the range between ~500 and 1500 nm, which exhibit a low absorption coefficient [26].

The advantage of using photonics as an external stimulus, in contrast to other physical stimuli like magnetic or acoustic ones, relies on the fact that photonic does not require expensive facilities or hard-to-use equipment; it is a low-cost therapy, besides being already comprehensively used in the clinical practice [25]. However, photons have limitations when it comes to their penetration in living tissues, which is considered a disadvantage. Water, molecules, cells, and biological structures present in living tissues interact with photons and usually display a high absorption coefficient. Nonetheless, the photon wavelength between 500 and 1500 nm, which corresponds to near-infrared (NIR), shows a low absorption coefficient in living tissues and is known as the therapeutic window (Figure 1) [26]. In this region, most photons can penetrate across the skin and reach other tissues. Despite their penetration, photons cannot reach deep cancer sites since light keeps being absorbed by biological components and water through the light path, which remains a limitation of photonic nanomedicine. In this sense, photonic nanomedicine can be applied only for selected cancer treatments, while other external stimuli-based nanomedicines, like magnetic hyperthermia, can be used for deeper cancers. That fact makes photonic nanomedicine a complement to other nanomedicines, and it does not eclipse other innovative therapies that have also been researched in the last few years.

In this work, we covered three prominent photonic nanomedicine therapies: (1) photothermal therapy; (2) photodynamic therapy; (3) photoresponsive drug delivery systems (DDS). These three therapies have been researched in the last years, attracting attention to their effectiveness and high temporal and spatial resolution. In the next sections, we shall review the advances in the aforementioned photonic nanomedicine therapies, focusing on the materials used, their advances, strengths, limitations, and perspectives.

## 2. Methodology

Aiming at covering the most recent advances in these three therapies, we established a methodology to carry out this review. A search was performed in PubMed and Web of Science databases, having “biomaterial” and “cancer treatment” as keywords. It was found that 12,937 works were published since the first years (PubMed, 1963 and Web of Science, 1945) of registers in these databases. Within the last five years (up to August 2020) 6142 papers were published. The search was then refined by choosing original contributions and important review articles containing any of these terms: photothermal therapy, photodynamic therapy, photosensitive hydrogel, photoresponsive hydrogel, light-sensitive hydrogel, light-responsive hydrogel, photonic nanomedicine.

## 3. Photothermal Therapy in Cancer Treatment

### 3.1. Background

Photothermal therapy (PTT) is an emerging cancer treatment that consists of light irradiation by a laser on a nanoparticle surface, maintained below the safe limit of tissue irradiation of 0.3–1.0 W/cm^2^ [27], which produces a localized surface plasmon wave acting directly on the tissue or photosensitizer agents brought to the excited state to release vibrational energy. Either plasmon wave or vibrational energy is converted into heat, thus causing hyperthermia (an increase in temperature of the surrounding tissue). Due to the acidic microenvironment of tumor cells, hyperthermia promotes effects such as protein denaturation, damage to the cytoskeleton, impairment of some DNA repair processes, changes in the permeability of the cell membrane, and stimulation of the immune system; however, surrounding healthy tissue and critical structures are preserved [28].

The heat generated above 37 °C leads to cell apoptosis, increasing the susceptibility of target tissues to other therapies, such as radiation and chemotherapy [29]. At 41 °C, changes in gene expression patterns occur [30]; temperatures below 42 °C (mild hyperthermia) promote an increase in blood flow to the tumor and, consequently, an increase in vascular permeability and the supply of oxygen and nutrients to the tumor cells [30]. However, at higher temperatures (the so-called extreme hyperthermia), tumor vessels collapse, leading to necrosis, apoptosis or coagulation, and hypoxia with irreversible tissue damage. When a moderate temperature is applied, oxygen consumption is reduced due to the directing of cell metabolism to the glycolytic pathway; 10 min exposure to a temperature of 42–46 °C leads to cell necrosis. Cytotoxicity, which is mild to moderate with the use of hyperthermia alone, increases rapidly when chemotherapy or radiation therapy is associated with PTT treatment [22,31]. Temperatures above 50 °C, called thermal ablation, directly destroy tumor cells; the use of PTT may help achieve tissue temperature above 60 °C, promoting protein denaturation and plasma membrane destruction, causing cell death almost instantly [27,30,32].

PTT is currently in the evaluation phase in multiple clinical trials. Research has been conducted to improve global therapeutic results for concomitant use of photothermal therapy with secondary strategies (chemotherapy, gene regulation, and immunotherapy). The heat generated by PTT can increase the permeability of tumor vessels to increase the accumulation of drugs and, combined with other therapies such as immunotherapy, can eliminate primary tumors and eventually disseminated metastases. Decreasing the required drug dosage also minimizes toxic side effects, improving the prognosis broadly [33].

Near infrared (NIR) light sources, also used in PPT, can be achieved using diode lasers (630–1100 nm), dye lasers (390–1000 nm), alexandrite lasers (720–800 nm), and neodymium-doped yttrium-aluminum-garnet lasers (Nd:YAG) (1064 nm), or by amplification or optical parametric oscillation [30]. Moreover, different optical apparatuses can be used to deliver light into the cancer site, such as frontal diffusing fibers for surface irradiation, multiple cylindrical diffusion fibers for interstitial light distribution in large-dimensional deeper tumors, and balloon catheters for irradiation of esophageal tissues [30,34,35].

Preclinical research focuses on the development of new photothermal contrast agents, pharmaceutical nanomedicines based on inorganic nanoparticles (noble metal semiconductors, metal-semiconductor, quantum dots, and metal oxide nanoparticles), plasmonic nanomaterials, semiconductor nanostructures (organic semiconducting polymer nanoparticles), and organic nanomaterials (organic dye molecules, organic nanoparticles, polymeric micelles, liposomes, dendrimers, nano-emulsions, and carbon-based materials, such as graphene oxide and carbon nanotubes, small molecules and semiconducting polymers) [34,36]. PTT agents can enhance the efficiency of localized light-based heating and ablation of tumor tissues and, at the same time, reduce adverse effects.

High-performance photo-converted energy-converting biomaterials (H-ECBs) produce heat to kill specific cells, not requiring oxygen to interact with the target cells or tissues, as in photodynamic therapy. The longer the light wavelength, the less energetic and less harmful to healthy cells and tissues surrounding the tumors [34]. Once irradiated, the PTT agents absorb photons’ energy, migrating from the ground singlet state to an excited singlet state. As it returns to the ground state, the so-called vibrational relaxation promotes collisions between the excited PPT agents which, in turn, collide with molecules surrounding their microenvironment. As a result, the temperature increases due to the increased kinetic energy [30]. Critical conditions for PTT success are the adequate spectral range, attaining the peak absorption wavelength of the photosensitive agent used in the treatment, and the tissue-penetration depth that can reach the target while reducing the power loss to a minimum [30]. When using PTT agents, the typical NIR range that excites PTT agents is 700–1000 nm (NIR-I) and 1000–1350 nm (NIR-II). NIR-II is less affected by scattering in tissues than NIR-I, improving treatment outcomes. Indeed, the limited penetration of light through biological tissues makes PTT, in general, ineffective for deep-seated tumors, making X-ray radiation or magnetic hyperthermia promising alternatives of energy source in such cases [30].

**Figure 2:**
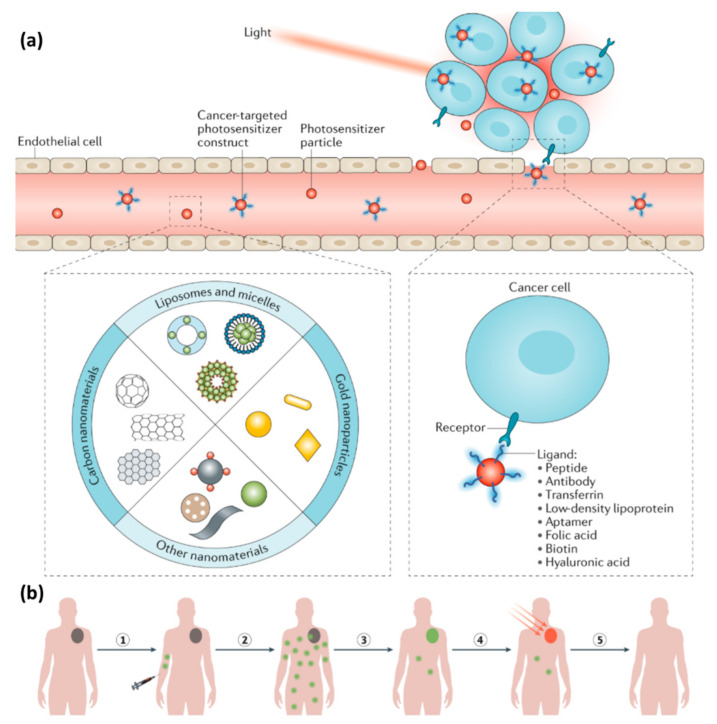
Use of nanoparticles and high-affinity ligands to target cancer and increase the spatial resolution of photothermal therapy (PTT): (**a**) nanoparticle accumulation in cancer cells through cancer-target ligands, where either the nanoparticles or the ligands can come from a wide range of materials; (**b**) scheme of drug accumulation in the cancer site—step 1 shows the nanoparticle injection, step 2 evidences the nanoparticle distribution in the human body, step 3 shows the nanoparticle accumulation in the tumor, step 4 highlights the photonic nanomedicine being used, and step 5 shows the complete tumor regression after the therapy. Copyright (2020) Springer Nature Limited. Source [30].

High-affinity ligands usually engage surface molecules, binding to receptors overexpressed by cancer cells or tumor epithelial cells. Peptides, proteins, aptamers, vitamins, and carbohydrates are classes of ligand being investigated for PTT active targeting [30]. Figure 2a presents the use of nanoparticles and ligands in PTT. The use of high-affinity ligands to target cancer cells improves the spatial resolution of PTT since it allows nanoparticle accumulation in the target site (Figure 2b).

### 3.2. Materials

#### 3.2.1. Inorganic Nanoparticles and Nanostructured Biomaterials

Inorganic nanoparticles consisting of noble metal-semiconductors, quantum dots, and metal oxide enable higher photothermal-conversion efficiency (PCE) and photo-stability than organic nanoparticles. However, low biodegradability limits their application in PPT [34]. Pt, Pd, Au, Fe, Ag, Ni, Cu, Se, or their oxides, and corresponding alloy, submitted to NIR light are known to convert it into heat via surface plasmon resonance (LSPR) [27,34]. Among these metals, gold is one of the most used in PTT preclinical research.

Typically, PTT is mediated by gold-based nanoparticles due to their biocompatibility, allowing the surface to be functionalized with molecules that increase the time of circulation (EPR effect), penetration, or both, into the tumor [33]. As nanoparticles, nanorods, nanocages, nanostars, nanocubes, and nanospheres, according to specific methods, Au-based PPT agents are biocompatible, promoting high stability in vivo, besides the adjustable absorption and excellent biosafety. Size and structure can vary according to the synthesis method. In a study evaluating Ag and Au nanoparticles obtained through bio-synthesis, it was observed that incubation with specific cancer cells allowed distinct cytotoxicity levels: specifically, Ag nanoparticles displayed higher toxicity than Au towards various cancer cell lines [33].

The high absorbance in the NIR-II region makes its photothermal performance remarkable, besides the potential for using it concomitantly with chemo-photothermal therapy in the NIR-II bio window [34]. Core-shell gold-silica nanoparticles have special functional groups responsible for additional multifunctionality, besides PTT applications [37]. The limitation remains for Au of poor photothermal stability. Since Pd and Pt are more photothermally stable than Au, developments have been conducted to enhance the PCE of noble metals [34].

Bioactive silicate glasses are mainly used in orthopedics and dentistry to restore small to mid-size osseous defects due to their capability to bond to living bone forming a strong interface and stimulate osteogenesis [38]. However, once adequately doped with small amounts of metallic cations, bioactive glasses can also exhibit valuable extra-functionalities [39]. In this regard, copper is a highly versatile dopant for bioactive glasses being able to elicit antibacterial and pro-angiogenic effects as well as suitability for use in photothermal applications [40]. Nanoporous sol-gel bioactive glasses have also been proposed in other strategies than photonic nanomedicine for cancer treatment (e.g., Fe-doped glasses for magnetic induction of hyperthermia) [41].

Polyoxometalates (POMs), a molecular group of polyanion inorganic clusters composed of transition metals bridged by oxygen atoms with precise chemical composition and architecture, assist in the detection of diseases and can act as a therapeutic element, synergistically, due to their redox absorption, being easily oxidized and reduced with no structural change. Acting as a multifunctional platform, they integrate not only PTT but also fluorescence detection, magnetic resonance, computed tomography, and photoacoustic images supported by photothermal properties. The heat derived from POMs upon light irradiation causes irreversible damage and death to cancer cells [42].

Special attention has been given to producing inorganic nanoparticles aimed at PTT and obtained through eco-friendly, green methods. Natural chemical synthesis of inorganic nanoparticles enables nanomaterials production without using toxic compounds, demanding raw materials easily found in nature, including plant metabolites, fungi, yeast, bacteria, viruses, and biopolymers, promoting a secured working environment in which biological materials are the precursors, and optimizing metallic ion bio-mineralization [27].

#### 3.2.2. Semiconductor Nanostructures

Metal semiconductor-based photo-converted hyperthermia-related energy conversion biomaterials (H-ECBs), as metal chalcogenides, metal oxides, and metal phosphides, have been used in PPT [34]. Regarding metal chalcogenides, copper chalcogenide-based photo-converted H-ECBs deserve special attention since this semiconductor promotes strong NIR-II absorption compared to other nanomaterials [34]. Concerning metal (Fe, Ni, Co, and Cu) phosphide-based photo-converted H-ECBs, such semiconductors have been successfully synthesized and completely ablated tumor cells under 1064 nm laser irradiation [23]. Nonetheless, regarding phosphite-based semiconductors, special attention must be given to tri-nickel monophosphide (NiP), one of the most prominent photothermal conversion materials, with an efficiency of 56.8%, surpassing other PPT agents based on phosphites, selenides, oxides, and carbides [43].

Besides nanoparticles, two-dimensional materials have also been addressed as prominent PTT agents. Two-dimensional (2D)-based photo-converted H-ECBs may be represented by 2D transition metal dichalcogenides (2D TMDs), such as MXenes, black phosphorus (BP), boron nanosheets (B), and reduced graphene oxide [44]. Among them, MXenes, including 2D transition metal carbides, nitrides, and carbon nitrides, demonstrated excellent biocompatibility and low toxicity, besides their large surface area, strong NIR absorption, and high electronic conductivity [30,45]. Similarly, black phosphorus nanosheets (BP) also display high biocompatibility and proper biodegradability for PTT applications [46].

#### 3.2.3. Organic Materials

Besides inorganic materials, organic nanostructures can also be designed for applications in PTT. Organic materials for PTT include organic dye molecules, organic semiconducting nanoparticles, polymeric micelles, liposomes, dendrimers, nanoemulsions, and carbon-based materials, such as graphene oxide and carbon nanotubes.

Organic semiconducting polymer nanoparticles are polymeric structures optically active with large π-conjugated aromatic or aromatic heterocyclic structures, include polyaniline (PANI), polypyrrole (PPy), and conjugated donor-acceptor (D-A) structures. The photothermal conversion of organic semiconducting polymer nanoparticles occurs under laser incidence in the NIR-II window range, which is more comprehensive than small molecules [34,47]. The decoration of nanoparticles with functional groups gives them targeting properties to achieve organs or tissues [37].

Cyanine, porphyrin, diketopyrrolopyrroles, phthalocyanine, and croconaine are part of the group of small molecular-based photo-converted H-ECBs [34]. Among the organic dyes, indocyanine green (ICG) is a contrast agent known to be useful for PTT in preclinical trials, requiring the use of 805 nm laser in preclinical tumor models. According to Dong et al. [34], cyanine molecules such as ICG, IR825, and IR780 are promising PTT candidates.

Carbohydrate polymers are stable, biocompatible, biodegradable, safe, non-toxic materials, representing suitable nanocarriers for PPT. However, most carbohydrate polymers cannot display photothermal conversion. In this sense, they can be mixed with photothermal agents, which are embedded within nanoparticle structures, such as multiwalled carbon nanotubes (MWCNTs) embedded in β-cyclodextrin (CD). When these nanocomposites are stimulated, they can flawlessly perform thermal ablation in the tumor microenvironment. Furthermore, chemotherapeutics can also be loaded in the nanoparticles in order to combine PTT with chemotherapy, and drug release can be performed over 30 h, according to findings from the literature [48].

Finally, another class of organic materials applied in PTT is graphene-based materials. Graphene oxide (GO) shows a high solubility feature in aqueous environments; however, it is unstable in the biological fluids containing salts, tending to adsorb proteins, making them vulnerable to macrophages eliminate them from the body. Moreover, their toxicity, low biocompatibility, and possible adverse side effects, depending on the dosage, limit their applications. Thus, functionalization with natural or synthetic polymers (even though natural polymers are often preferred due to higher biocompatibility, biodegradability, and lower toxicity) are strategies able to improve their use without the adverse effects, enhancing GO properties [36]. Reagents prone to functionalize graphene are N-hydroxysuccinimide (NHS), 1-ethyl-3-(3-dimethyl aminopropyl) carbodiimide (EDC), and thionyl chloride (SOCl), leading to imide or acyl chlorides groups formation, preserving or controlling chemical and electrical properties of graphene due to covalent modification of its surface, not changing its structure. Besides, grafting or functionalizing graphene-based materials enables the coupling of PTT with drug delivery systems, where drugs can be bonded or trapped within the grafting or functionalizing structure. Moreover, functionalization with polysaccharides provides water solubility, biocompatibility, stability, bioavailability, enhanced loading capacity to the nanocarrier while minimizing immune system activation [36].

### 3.3. Applications

Clinical research in laser ablation without PTT agents has already been extensively used in clinical trials [30,36]. The review conducted by Datta et al. (2020) [49] list a series of cancer types susceptible to treatment with hyperthermia, including, but not restricted to various superficial tumors, melanoma, choroidal melanoma, brain tumors, malignant germ cell tumors, soft tissue sarcoma, bone metastases, locally advanced head, and neck cancer, cancers of the esophagus, breast, lung, pancreas, urinary bladder prostate, rectum, anus, and pelvis, and other tumors.

Among the comprehensive, diverse class of materials used in PTT, gold nanosheets are among the most advanced materials concerning the different steps towards clinical use. Actually, PEGylated silica-cored Au nanoshells were the first photothermal nanoparticles to have advanced into clinical trials, produced by the company Nanospectra Bioscience (Houston, TX, USA), and commercially named as AuroShell (Houston, TX, USA) [50]. The therapy using these nanoparticles is called AuroLase therapy and is under clinical trials for primary and metastatic lung cancer [17,51]. A successful result from these clinical trials is expected once these nanoparticles show high enhanced permeability and retention effect, which enables their accumulation in tumors. Furthermore, a recent clinical pilot study showed a 94% efficacy of photothermal ablation using a gold nanosheet to treat prostate cancer [52]. Altogether, these studies highlight the excellent use PTT agents in cancer treatments.

Among the organic materials used in PTT, indocyanine green (ICG) is one of the most researched materials. The main advantage of using organic materials like dyes is their small size, making them easy to combine with other materials and produce multifunctional materials. For example, recently, Wen et al. [53] designed and synthesized hydrogen-peroxide-responsive protein biomimetic nanoparticles (MnO_2_-ICG@BSA) for melanoma treatment using PTT-PDT therapy. The authors used a mild protein synthesis method, in which ICG was loaded into a bovine serum albumin-manganese dioxide complex (MnO_2_@BSA), achieving high photothermal conversion efficiency and high photothermal stability, besides low toxicity observed in preliminary toxicity evaluations. Besides Au-based nanoparticles and ICG, most other inorganic and organic PTT agents are still undergoing pre-clinical studies.

PTT agents can also be allied with regenerative materials, focusing on applications as theragenerative materials, which combine therapy with regeneration [54]. An interesting example is the applications of bioactive glass scaffolds with photothermal conversion properties. These materials can treat bone cancer by PTT, as well as regenerate the bone loss caused by the tumor. Yu et al. [40] incorporated copper into hollow silicate glass microspheres to elicit multiple therapeutic actions, i.e., uptake and delivery of an anti-cancer drug (trametinib) used in chemotherapy allied with PTT of skin cancer, along with stimulation of skin tissue regeneration. In another study, Liu et al. [55] used 3D printing to prepare Cu/Fe/Mn/Co-multidoped bioactive glass-ceramic macro-nanoporous scaffolds with photothermal effect, besides stimulating osteogenic differentiation. These multidoped bioactive scaffolds, containing up to 5% dopants, generally exhibited good photothermal activity, and their performance followed the trend 5Cu- > 5Fe- > 5Mn- > 5Co-doped glass-ceramic. Moreover, the hyperthermic effect generated by 5Cu-, 5Fe- and 5Mn-containing samples could effectively kill bone tumor cells in vitro (Saos-2 cell line) and inhibit tumor growth in vivo (rat model). More specifically, 5Fe- and 5Mn-doped samples were suggested as promising candidates for PPT of bone tumor and bone regeneration since they showed a better substrate for adhesion of mesenchymal stem cells. Regarding organic dyes, ICG has shown noticeable results in treating advanced-stage metastatic breast cancer, with no serious adverse effects [56].

### 3.4. Strengths, Limitations and Perspectives

Light sources comprehend infrared and laser infrared (IR) heating lamps (frequency > 300 GHz). However, due to O–H bonds from water molecules of living tissues, energy is strongly absorbed. Therefore, PTT has restrictions in penetration depth, which does not exceed 1 cm [35]. Thus, PTT use for superficial cancer tumors as a breast cancer treatment is possible [34]. Non-specific overheating of the surrounding healthy tissue when using laser ablation is one of the most critical limitations [28]. Drawbacks of PTT for the complete elimination of solid tumors stimulate therapy combinations, exploring synergistic therapeutic effects, avoiding MDR and hypoxia-related resistance are frequently seen in cancer therapies [23]. Combinations comprise PTT and photodynamic therapy (PDT), or PTT and PDT with chemotherapy or immunotherapy. In the first case, as the light heats the tissues, blood flow increases at the light-irradiated site, increasing the tumor’s oxygen content, enabling reactive oxygen species (ROS) formation and, consequently, PDT efficiency.

Regarding nanoparticles used in PTT, although they can benefit from the EPR effect, some tumors in the initial stages have irregular vasculature, decreasing nanoparticle intake by cancer cells. However, this limitation can be overcome by using high-affinity ligands that selectively bind to cancer cells. Inorganic nanoparticles and nanostructured biomaterials, even if inorganic nanoparticles result in greater photothermal conversion efficiency (PCE) and photo-stability than organic nanoparticles, a limitation to their application in PPT lies in their low biodegradability [34]. However, gold nanoshells have been considered a promising contrast agent in preclinical trials in prostate, head, and neck cancer tumors, even though results were considered less efficient than PTT with other contrast agents [19,22]. Moreover, the synthesis, heterogeneity, modulation of properties, surface modification, targeted toxicity, imaging, and bio-detection potential of manganese oxide nanomaterials (MONs including MnO_2_, MnO, Mn_2_O_3_, Mn_3_O_4_, and MnO_x_) and their derivatives have also shown significant progress [57]. Very recently, some multicomponent oxide-based bioactive glass compositions have also shown promise for application in cancer treatment via PTT. On the other hand, organic nanoparticles’ toxicity, low biocompatibility, and possible adverse side effects depending on the dosage, as previously mentioned, limit their appliance. Functionalization with natural or synthetic polymers configures strategies to avoid adverse effects [36].

Organic nanoparticle application in PTT appears as a promising strategy. The biological characterization of nanoparticles produced through biosynthesis has already brought exciting results. Due to the union of proper biological properties and high-efficiency photothermal conversion, semiconductor nanostructures as BP have recently gained attention as prominent PTT agents [58]. Also, given the anti-tumor and immunological effects derived from hyperthermia, organic dyes-based PTT with immunotherapy has become an important research area [53].

The combination of PTT with PDT is another promising trend in cancer treatment, and valuable results have been shown in the literature. For example, human hepatoma cell line (HepG-2) was treated in vitro to understand the therapeutic efficacy of GFCDH nanoparticles (cystine-functionalized disulfide bonds bonded graphene oxide (GO-SS), coating folic acid (FA) conjugated chitosan (CS) based-cores (FCDH)) combined, among other techniques, with photothermal therapy and photodynamic therapy [59]. The cytotoxicity results showed highly toxic against human hepatoma cells (HepG-2) induced by 808 and 700 nm light, in synergistic effect with PTT, demonstrating potential application of multi-responsive nanosystems in cancer treatment [59].

## 4. Photodynamic Therapy Applied in Cancer Treatment

### 4.1. Background

Photodynamic therapy (PDT) has been successfully employed in cancer treatment due to its ability to kill cancer cells through a localized generation of oxidative stress, preserving normal tissues [60]. It employs three individually distinct agents: the photosensitizer (PS), which is a photoactivatable drug, light (mainly lasers) [61], and molecular oxygen (O_2_) [62]. During PDT, the cytotoxic species singlet molecular oxygen (^1^O_2_*) and ROS are generated from a photodynamic process involving energy transfer from the PS in the triplet excited state to the ground state (O_2_) upon light exposure. Both ^1^O_2_ and ROS are very reactive molecules, with a short half-life, affecting a radius of 20 nm, limiting the oxidative stress to the site of the application (tumor tissue) and preserving the adjacent normal tissue [63]. The photo-generated species have toxic effects like killing cancer cells by oxidizing vital nucleic acids, proteins and lipids, or promoting a death signaling cascade [64]. In this sense, in situ generated singlet oxygen promotes apoptosis, necrosis, activation of the immune system, macro-autophagy, and tumor vasculature destruction. An Acute inflammatory process is induced by PDT, which triggers the release of cytokines leading to cell death. Moreover, a high influx of leukocytes strongly contributes to tumor destruction [65]. Ideally, PS is not toxic in the dark condition, and its physical interaction with light at the tumor site produces local cytotoxicity. For successful therapy, nontoxic PS selectively localizes in solid tumors and tumor vasculature. Thus, PS localization is of fundamental importance in the success of PDT. In general, PDT is dependent on the chemical nature of PS, dosage, light source/intensity, and exposure time [62]. Different kinds of PS have been employed nowadays, allowing them to have affinity for different organelles/parts of the cells. Upon the irradiation of light at the tumor site, PS interacts with light, leading to ROS and single oxygen in the affected tissues, preserving normal tissues [65]. In PDT, light has a suitable wavelength and energy, usually laser, in addition to other sources [66]. Usually, in PDT, the light wavelength ranges from 600 to 800 nm, including blue, red, and infrared lights. The right choice of light source depends on the nature of the tumor, chemical nature of PS (its absorption spectra), location and size [62].

There are several kinds of PS available for PDT, and the first PS approved by the Food and Drug Administration (FDA) was the hematoporphyrin oligomer (Photofrin, Bannockburn, IL, USA), which has been extensively employed for clinical use since 1996 in different solid tumors, such as brain, breast, bladder and prostate malignancies [64]. Due to its feature, PDT has been considered a smart approach for cancer treatment. The use of PDT is common in in vitro, in vivo, and some clinical trials. This technique has also been approved for treating severe solid cancers such as lung, melanoma, bladder, cancer of the esophagus, and topical lesions [67]. The main advantage of PDT in cancer is treating the tumor tissue with minimal side effects than traditional therapies [64]. Interestingly, PDT can be employed in cancer treatment before or after radiotherapy, chemotherapy, or surgery, suggesting its versatility [62].

### 4.2. Materials

Although PDT is considered a promising therapeutic tool in the treatment of solid tumors, due to the complex biological responses/interactions and cell signaling, it has been reported that cancer cells might acquire cellular resistance to PDT by modulating photosensitization/PS action. Essentially, the reported cell resistance mechanisms to PDT are complex and depend on the PS. PDT can be almost ineffective in some instances due to cell resistance, as observed in traditional chemotherapy [68]. In addition to the expected and desired direct cellular toxicity caused by PDT, several studies have reported the effects of this therapy on cell signaling and gene expression. Indeed, PDT can activate cell signal transduction pathways and the expression of signal-regulated kinases [69]. Antiapoptotic Bcl-2 proteins can be activated by PDT [70], as well as the autophagic response of cells [71].

In order to overcome the cancer cell resistance mechanisms to PDT, the combination of PDT and nanotechnology has been extensively studied as a promising approach for enhancing PDT while minimizing/avoiding cellular resistance [65,72]. Fundamentally, nanotechnology has been successfully applied to efficient PS modification through its functionalization or conjugation with engineered nanoparticles and other active compounds, such as immune agents. Association/modification of PS and nanocarriers might allow a direct and efficient PS delivery to the target site (tumor tissues). Smart and versatile nanomaterials have been designed not only as useful carriers for PS delivery but also as photoactive agents due to their chemical features. The combination of PS and nanomaterials can destroy tumor cells with minimum side effects to normal cells due to the site-specific property of the engineered nanoparticles. It should be noted that to combat resistant tumors, the increase of the dose of the PS or the irradiation time should not be explored to avoid PS uptake by normal cells [65]. Efficient PS uptake by cancerous cells is an essential prerequisite for successful PDT, which can be enhanced by PS conjugation with nanomaterials, avoiding non-specific PS distribution in the body and minimizing the side effects.

In this direction, several nanomaterials have been designed as drug delivery systems. Quantum dots, liposomes, metal oxide nanoparticles, polymer dots, and nanotubes are the most common nanocarriers used for PS delivery to cancer cells [73]. These nanomaterials have higher cross-sections for absorbing light in comparison with PS in the bulk state. Some nanomaterials can be designed to absorb light with different wavelengths ranging from near UV to near infra-red [73]. The nanomaterial choice depends on many parameters, such as the kind of tumor, the chemical nature of PS, the desired therapeutic effect, cost, thermal stability, etc. [74]. Conjugation of PS into a nanomaterial can protect PS against enzymatic and/or thermal degradation, promote a sustained PS release from the nanomaterial direct to the desired site of treatment (cancer cells), facilitate the uptake of PS by cancer cells, and allow the delivery of other active drugs in combinatory cancer therapy.

**Figure 3:**
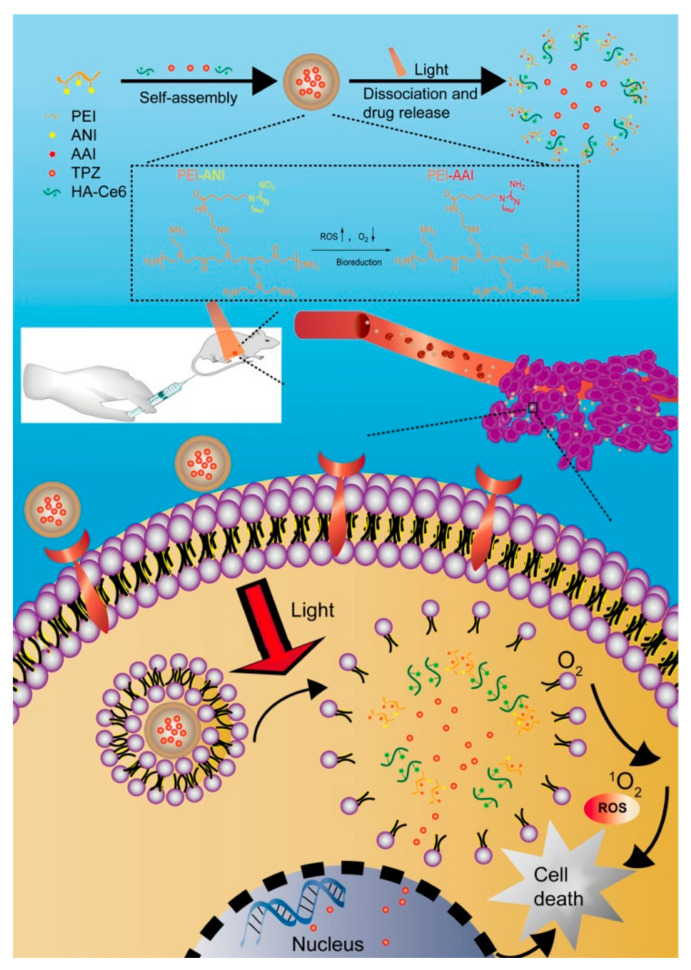
Schematic representation of the dual hypoxia-responsive amphiphilic polyethyleneimine−alkyl nitroimidazole (PA)/hyaluronic acid-chlorin e6 tirapazamine (HA-Ce6@TPZ) nanoparticles toward photodynamic therapy (PDT)-strengthened bioreductive therapy. Source: reprinted with permission from [75]. Copyright (2019) American Chemical Society.

For instance, a nanomaterial was developed to allow PDT-induced drug release and drug activation by hypoxia [75]. Self-assembled nanoparticles composed of amphiphilic polyethyleneimine-alkyl nitroimidazole [PEI−ANI, (PA)] and hyaluronic acid-chlorin e6 (HA-Ce6) were prepared to encapsulate the chemotherapeutic tirapazamine (TPZ) efficiently. Upon a systemic administration, the engineered nanoparticles accumulate into the tumor tissue due to the HA-mediated cancer target. After endocytosis by cancer cells, high ROS levels are locally produced upon irradiation with light (600 nm, 10 mW/cm^2^), generating local hypoxia leading to the NP degradation and the release of the active drug (TPZ). Under hypoxia, TPZ is activated, allowing a potent synergistic anticancer effect (Figure 3). This study elegantly illustrates a versatile and suitable approach to use nanotechnology in combination with PDT.

Altogether, recent progress in nanotechnology has been promoting a positive impact in PDT for cancer treatment, creating new versatile and smart engineering nanomaterials as vehicles for PS, and further studies are required in this exciting field of research.

### 4.3. Applications

The endogenous free radical nitric oxide (NO) is an important signaling molecule that modulates several physiological and pathophysiological processes [76]. The NO is a gaseous free radical, and NO donors have been used as pharmacological agents in cancer treatment. At low concentrations (pico-nano molar range), NO controls physiological processes, such as the promotion of blood flow, iron homeostasis, and neurotransmission. NO has toxic effects at high concentrations (micromolar range), acting as a defender against pathogens and tumors [77]. Critical studies have described the use of NO/NO donors in cancer treatment to promote direct toxic effects on tumors and induce sensitization of cancer cells, thereby mitigating and even reversing the MDR observed in some tumors [78]. Significantly, MDR of cancer cells can be reversed by NO via reduction of P-gp expression levels, thus sensitizing cancer cells to therapies, including PDT [79]. Recently, the combination of NO/NO donors and nanomaterials has been emerging as a potent approach to promoting the sustained release of NO directly to the tumor tissue. In this scenario, the development of smart nanomaterials able to release NO under controlled conditions, such as light irradiation, has been the focus of intensive research. NO therapy in cancer cells has been considered a “green” treatment due to minimum side effects to normal tissues [76]. NO can be generated by thermal or photodecomposition of precursors when accumulated in solid tumors [80]. Thus, the development of biocompatible nanocarriers able to allow the controlled release of therapeutic NO amounts directly to the desired site of application (tumors) combined with other chemotherapies, such as PDT, is at the forefront of biomedical research in this field [77].

**Figure 4:**
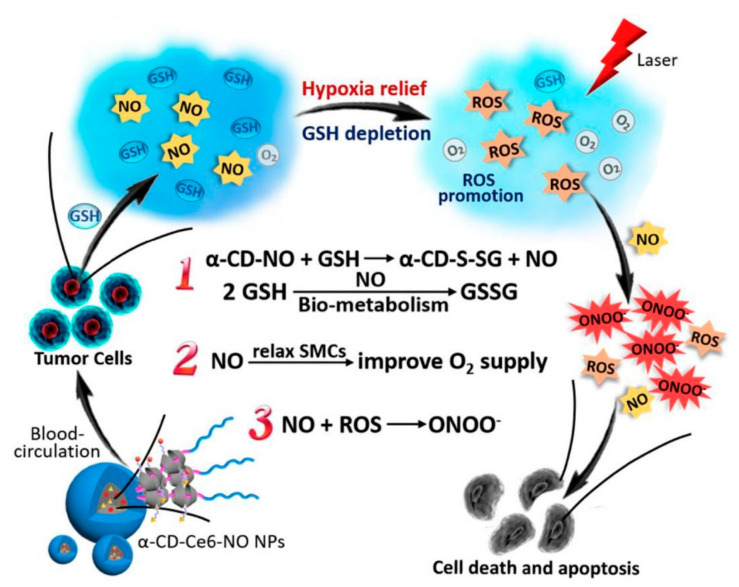
Schematic representation of multiple synergistic effects between PDT and nitric oxide (NO) generated from the nanoparticles α-cyclodextrin-chlorin e6-NO (α-CD-Ce6-NO) nanoparticles (NPs) improves anticancer efficacy. Reproduced with permission from Elsevier. Source: [81].

Several works report the combination of NO donors with PDT in the fight against cancer. A NO-releasing nanogenerator allied with PDT was developed by integrating glutathione (GSH)-NO-prodrug into a nanomaterial (Figure 4) [81]. This nanomaterial can deplete intracellular GSH and relieve hypoxia in the tumors through NO generation since NO is a vasodilator. The toxicity to tumor cells was enhanced by the production of reactive nitrogen species (RNS) generated from the reaction of NO and ROS derived from α-cyclodextrin (α-CD) conjugated S-nitrosothiol (NO donor) and laser light-activated chlorin e6 (Ce6) (PS) (Figure 4). In this smart approach, NO acts not only as a toxic agent against solid tumors but also in the amplification of the therapeutic effects of PDT. NO is known to react with ROS producing the harmful peroxynitrite (ONOO^−^) that damages the cancer tissue. Interestingly, at the tumor site, NO promotes vasodilation, and thus, reducing hypoxia enhances the action of PDT via synergistic effects [81].

The combination of photo-release NO from lipid-polymer hybrid nanoparticles and doxorubicin (DOX) was recently reported to overcome DOX resistance in cancer cells. NO release was controlled upon visible light irradiation and reported potent toxic effects against DOX-resistant melanoma cells [82]. In a similar approach, a nanomaterial comprised of a NO donor (*N*-nitrosated napthalimide (NORM)) and tetraphenylporphyrin (TPP)-modified hyaluronic acid nanoparticles for the generation of peroxynitrite via interaction of NO and PDT was reported [83]. Upon irradiation with 365 and 650 nm light, NO is released from the nanoparticles and reacts with superoxide anion radical (O_2_^−^) yielding the biocidal molecule ONOO^−^.

### 4.4. Strengths, Limitations and Perspectives

As stated before, recent progress in the literature has shown that the combination of NO donors, PS, and nanomaterials is opening new venues in multimodal anticancer treatment, with features able to overcome the major issues found in traditional chemotherapy [83]. Further studies are required in this field. PDT allied to nanomaterials might minimize cellular resistance of cancer cells, and this effect opens new avenues in this field of research.

The significant advances of this therapy can be summarized as: (i) possible synergistic effects by combining PDT and nanomaterials containing active agents, such as NO donors, optimizing the anticancer effects while minimizing possible side effects, (ii) depending on the nature of the nanomaterial and the tumor, an ability to promote a toxic effect directly on the target site of application (tumor tissues), not affecting the normal tissue/organs, (iii) an ability to combine different engineered nanoparticles, with different PS and active chemotherapeutic drugs, under selective wavelength for treatment enhancement.

Although noticeable progress has been achieved using PDT in cancer treatment, some critical issues still need to be further investigated to allow successful clinical translation and future commercialization. In this sense, the key areas that deserve further investigation are in vivo studies to better evaluate the efficacy of the treatment and possible side effects and toxicity. To this end, pharmacokinetic and pharmacodynamics studies are essential. Furthermore, the fate and the toxic effects of the materials after the treatment (light application) should be carefully addressed.

## 5. Photoresponsive Hydrogels in Cancer Treatment

### 5.1. Background

Drugs can be defined as chemical compounds aimed to promote, relieve, or treat diseases. Usually, drugs act on cell signaling mechanisms, up or downregulating them to cause the desired effect [84]. The performance of a drug is evaluated under two main factors, pharmacokinetics and pharmacodynamics. Both factors are related to the stages of drugs after administration. Pharmacokinetics evaluates a drug concentration and kinetics in the bloodstream, which covers studies like drug absorption, distribution, metabolism, and excretion. In contrast, pharmacodynamics studies drug effects in the body, establishing a correlation between the dose taken and its concentration in an organism over time [85].

Although drugs can be designed to act on specific cells, organs, or tissues of an organism, putting together spatial and temporal resolution is challenging, despite the efforts of pharmacokinetics and pharmacodynamics studies to understand how a drug accumulates and is metabolized in the drug target. Moreover, a drug may display either medicinal or toxic effects depending on its concentration in an organism. This concentration interval is known as the therapeutic window. The therapeutic window is limited by two concentrations: (i) the minimal effective concentration (MEC); and (ii) the minimum toxic concentration (MTC). Once a drug is maintained in the therapeutic window, it is expected to bring the expected biological effect, with minimal or no side effects [86].

However, keeping the drug concentration within the therapeutic window is quite challenging since the drug cannot be suddenly released, which would cause a burst effect, nor be at a too low concentration unable to reach the MEC. Then, drugs are usually mixed with other chemical substances—producing pills, gels, solutions for injections—which have a controlled degradation rate that allows the drug to be delivered over a controlled release, i.e., in a time-dependent manner [87].

Recently, hydrogels have attracted attention as prominent vehicles for drug delivery since these materials are biocompatible in different tissues, increasing their application in pharmacology [88]. Hydrogels are defined as materials with a polymeric structure containing hydrophilic groups capable of holding a large volume of water in a three-dimensional network [89]. The ability to hold a high volume of water is derived from hydrophilic groups found in the polymeric chains, like –OH, –SO_3_H, –CONH, and –CONH_2_. Therefore, hydrogels can be made of different materials, such as natural polymers (collagen, hyaluronic acid, chitosan, alginate) or synthetic polymers—poly(vinyl alcohol), poly(hydroxyl methacrylate), poly(ethylene glycol) [90]. Moreover, these hydrogels can be found in different morphologies, such as three-dimensional macroscopic gels, or as nanostructures like micelles or nanocapsules [91]. Then, bioactive molecules can be loaded into their structure and be stored in the hydrophobic or hydrophilic portions depending on their nature.

Besides these mechanisms mentioned above, hydrogels can be designed to display stimuli-responsive properties, like thermo-responsive, pH-responsive, photoresponsive, electric-responsive, and magnetic-responsive. In these cases, the hydrogel is naturally able to self-assembly into ordered structures upon an external stimulus, or stimuli-responsive moieties are introduced in the hydrogel structure and modulates their self-assembly ability [88,92]. In the case of stimuli-responsive hydrogels, the main advantage is drug release allowance only after the stimulus (temporal resolution); thereby, the stimulus can be applied after the drug delivery system reaches the target tissue (spatial resolution) [87]. In this review, we shall focus on photoresponsive hydrogels.

Among the different stimuli-responsive hydrogels available for controlled release technology, photoresponsive hydrogels have attracted attention since light stimulus can be localized in time and space, and the light stimulus is triggered from outside the patient [93,94]. Therefore, in cancer treatments, photoresponsive hydrogels can deliver chemotherapeutics, immunotherapeutics, or other therapeutics into the cancer site, bringing a more effective localized treatment.

### 5.2. Materials

In general, photoresponsive hydrogels are based on, but not limited to, block copolymers, which consists of at least two blocks, where one is hydrophobic and another is hydrophilic. When these block copolymers are dispersed in water, they self-assemble into micellar structures if a critical concentration is reached [95], thereby forming a hydrophobic core surrounded by a hydrophilic corona. Then, these micelles can be loaded with active hydrophobic molecules in the core, or hydrophilic ones in the corona structure [96]

In order to make these hydrogels photoresponsive, there are two main strategies: (I) introducing photoreactive moieties into hydrogel polymers [96,97]; (II) producing hydrogel composites containing photothermal transducing agents (PTA) [98]. For each strategy, there are some limitations, challenges, and advantages.

#### 5.2.1. Drug Delivery Systems Based on Hydrogels Containing Photoreactive Moieties

Photoreactive moieties, also known as photoresponsive moieties or photoresponsive groups, are usually photochromic chromophore structure, which converts a photoirradiation into a chemical signal like photocleavage, photoisomerization, and photodimerization [97].

Photoisomerization reactions are often repeatable and reversible processes and consist of a trans to cis or cis to trans isomerization induced by photoirradiation. Some examples of photoisomerization moieties are azobenzene and spiropyran. When these moieties are in the cis configuration, they display higher polarity than the trans configuration due to molecular and electron cloud arrangement. Thus, these moieties are added to the polymer structure, making the polymer more hydrophilic in the cis configuration and more hydrophobic in the trans configuration [99].

Photocleavage reactions may be either reversible or not and consist of a chemical bond cleavage after photoirradiation. Examples of photocleavage moieties are o-nitrobenzyl and triphenylmethane. However, o-nitrobenzyl is a non-reversible photocleavage group, while the triphenylmethane dissociates into a pair of triphenylmethyl cation and hydroxyl anion; the triphenylmethyl is also photoreactive and associates with hydroxyl under photoirradiation, making the triphenylmethane a photoreversible moiety. In this case, the cationic state of o-nitrobenzyl and triphenylmethyl can be used as hydrophilic moieties and their neutral state as hydrophobic moieties [100]. Other less common photocleavage moieties, but also used in hydrogels, include azosulfonates, diphenyl iodonium-2-carboxylate, and phenylmethyl ester [97].

Photodimerization consists of a dimerization reaction induced by photoirradiation. The most known example is coumarin, which can undergo photodimerization, and the resultant dimer can suffer photocleavage, which makes coumarin a photo-reversible moiety. By controlling the crosslink bond density of coumarin moieties, it is possible to control the gelation and swelling of polymers, a different mechanism from the other photoreactive moieties [101].

**Figure 5:**
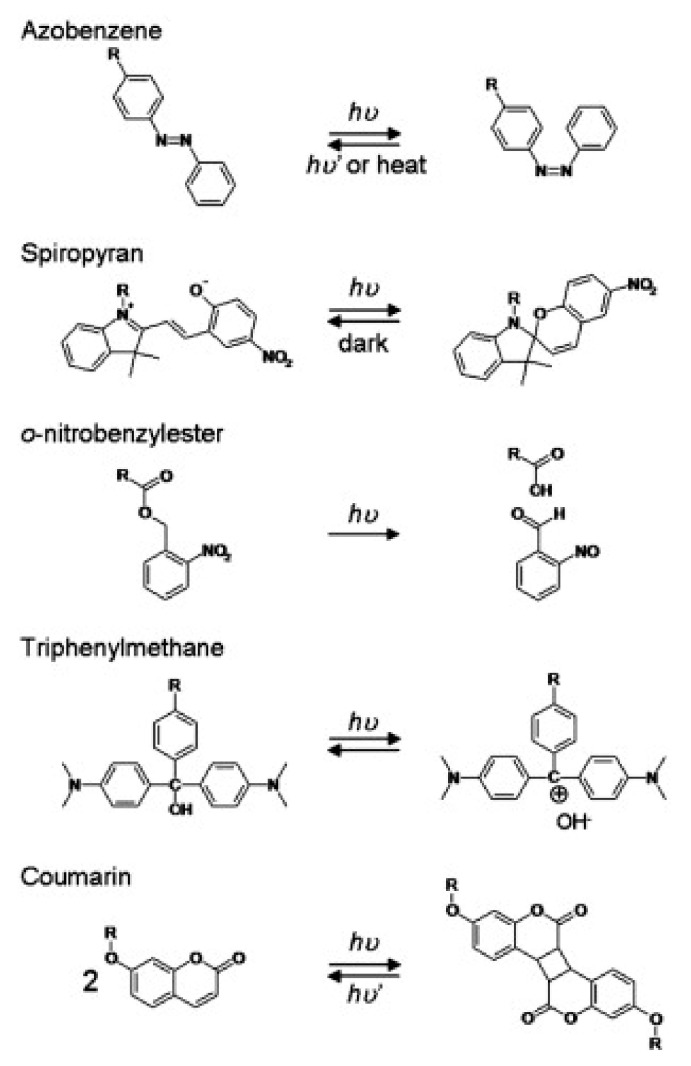
Representative photoreactive moieties used in photoresponsive hydrogels systems. Copyright (2011) Elsevier B.V. Source: [97].

Some examples of photoreactive moieties are shown in Figure 5, including photoisomerization, photocleavage, and photodimerization.

**Figure 6:**
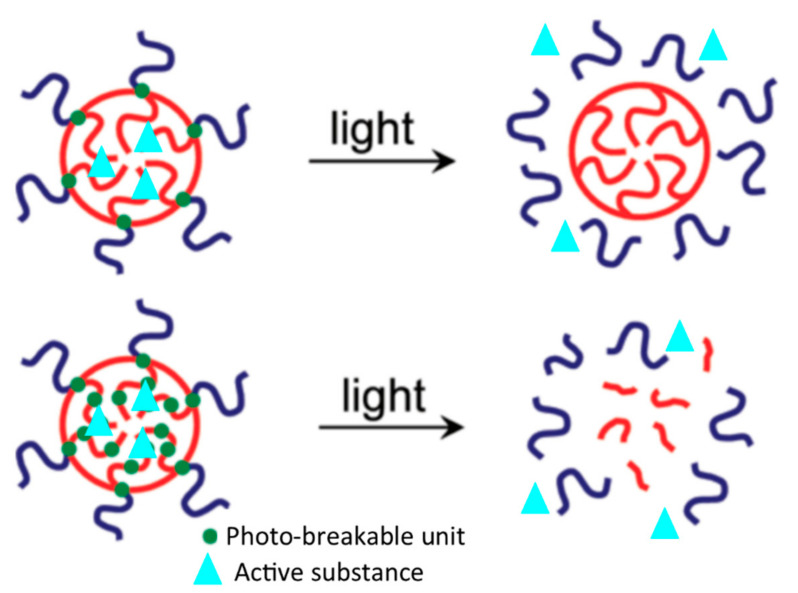
Representative figure of the mechanism of drug release from a light-responsive hydrogel: photoreactive moieties placed at the side group (upper example); photoreactive moieties placed at the main chain (lower example). Source: adapted with permission from [103]. Copyright (2011) American Chemical Society.

In order to make a hydrogel photoresponsive, the photoreactive moieties can be introduced in the polymer chain in two different configurations: (a) as a side group; (b) or in the main chain (see Figure 6). In hydrogels modified with photoresponsive moieties as side groups, usually, photoreactive moieties are bound to poly(methacrylate) block (PMA) in an ester bond [96,102], while the other block is composed of a hydrophilic polymer like poly(ethylene oxide) (PEO). Therefore, these block copolymers can form micelles consisting of a PMA core and PEO corona, suitable for carrying bioactive molecules into target tissues. Because the photoreactive moieties are in the hydrophobic core, when the micelles are photo irradiated, the photoreactive moieties shift from the neutral or hydrophobic configuration to the hydrophilic one. This process makes the two blocks hydrophilic, causing the micelle to collapse and allowing drug release from the micelle [97].

In hydrogels containing photoreactive moieties in the main chain, these moieties are included in the hydrophobic chain. Usually, the hydrogels are composed of a PEO hydrophilic block bonded to a hydrophobic poly(methacrylate) block [103]. Therefore, the photoreactive moieties are located in the core of the micelles. When the micelles are photo-irradiated, the photoreactive moieties become hydrophilic, leading to the micelle structure’s disruption [96,104].

#### 5.2.2. Drug Delivery Systems Containing Photothermal Transduction Agents

Photoresponsive hydrogels do not necessarily need to be based on photoreactive moieties, but they also include the development of composites consisting of a thermoresponsive hydrogel and a photothermal transduction agent (PTA). Unlike photoreactive moieties that convert photon energy into chemical reactions, photothermal transduction agents convert photon energy into thermal energy. Thereby, when the PTA is mixed with thermoresponsive hydrogels, they can modulate the hydrogel gelation due to the release of heat that is later absorbed by the surrounding medium.

There are different sorts of thermoresponsive hydrogel, but they can be mainly classified into natural or synthetic ones. Regarding natural thermoresponsive hydrogels, chitosan, cellulose, and gelatin/collagen stand as the more known polymers. On the other hand, synthetic thermoresponsive hydrogels are mostly based on poly(N-isopropylacrylamide) (PNIPAAm) and Pluronic poloxamer (Sigma-Aldrich, St. Louis, MS, USA). Regardless of being natural or synthetic, most of both kinds of thermoresponsive hydrogels display low critical solution temperature (LCST). These hydrogels are highly hydrated at a lower temperature, but when the temperature is above LCST, the water moves into a bulk solution, causing the collapse of the hydrogel itself, leading to hydrophobic interactions [105].

Natural polymers can form hydrogels because they have hydrophilic moieties in their structure, enabling polymer swelling in aqueous environments [105]. However, their thermoresponsiveness is modulated by different features. For example, chitosan, cellulose, and gelatin hydrogels are usually thermoresponsive only after grafting or copolymerizing it with hydrophilic chains or moieties like poly(ethylene glycol) N-isopropyl acrylamide (NIPAAm), and poly(acrylic acid) [105,106]. Cellulose can also become thermoresponsive after methylation, forming methylcellulose or carboxymethyl cellulose [107]. Gelatin can also be naturally thermoreversible since it is derived from collagen, thereby above 30 °C, the triple helix structure becomes less rigid, which changes the hydrogel’s ability to hold water [108].

Regarding synthetic thermoresponsive hydrogels, PNIPAAm is a hydrophilic polymer able to store a large amount of water due to polar moieties found in the N-isopropyl acrylamide, thereby having the properties needed for a hydrogel. Below the LCST, the PNIPAAm assumes a coil conformation, while above LCST, the hydrogel suffers a volume phase transition due to loss of hydrogen bonds. Then, hydrogel becomes more hydrophobic, which also leads to its collapse. After collapsing, the hydrogel assumes a globular structure [109]. The advantage of PNIPAAm is that these thermal transitions occur near 32 °C, which is close to body temperature. Then, drugs can be loaded into the PNIPAAm structure and be release after hydrogel collapsing. Therefore, PNIPAAm becomes a promising candidate as a hydrogel for biomedical applications [110].

Pluronics or poloxamers are triblock copolymer composed of poly(ethylene oxide)-b-poly(propylene oxide)-b-poly(ethylene oxide) (PEO–PPO–PEO). These triblock copolymers self-assembles into micelles in aqueous solution at pH and temperatures near-physiological conditions; this micellization occurs because PPO and PEO become less hydrophilic near 37 °C, lowering the energy to form micelles due to a gain in entropy. However, below body temperature (~37 °C), these micelles are not well structured in solution, while at body temperature, the micelles stack into ordered structures, forming a gel phase [110]. This process is known as the sol-gel transition. Once drugs are incorporated in the micelles in the sol phase, they can be released according to controlled kinetics when the hydrogel is in the gel phase. The release often occurs due to erosion and dissolution of the hydrogel, as well as drug diffusion mechanisms [88].

Regarding the materials used as photothermal transductors agents, usually, they are the same as those used in PTT, e.g., inorganic nanostructures of gold and iron oxides [22,31], and organic nanocomposites of polypyrrole, polyaniline, carbon nanotubes, and graphene analogs [23,111,112,113].

### 5.3. Applications

**Figure 7:**
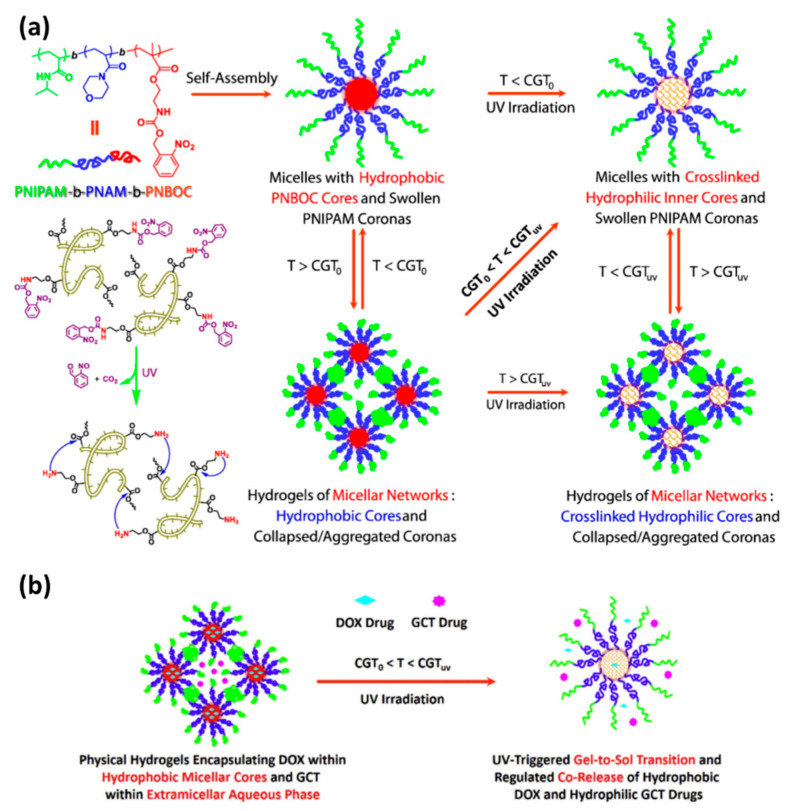
An example of a complex photoresponsive hydrogel: (**a**) schematics of temperature and ultraviolet (UV)-responsive micelles and supramolecular structures upon different conditions of temperature (T), critical gelation temperature before (CGT_0_), and after (CGT_UV_) UV irradiation; (**b**) scheme of UV-triggered gel-to-sol transition and co-delivery of doxorubicin (DOX) and gemcitabine (GCT) release. Copyright (2016) Elsevier B.V. Source: [94].

In the last decade, the development of drug delivery systems using hydrogels as vehicles has grown significantly. It has consisted of very elegant and complex systems, which sometimes combine more than one stimuli-responsive response in the same drug delivery system. For example, Wang et al. [93] developed photo- and thermal-responsive multicompartment hydrogel to deliver hydrophilic and hydrophobic drugs synergistically. The hydrogel was based on an amphiphilic triblock copolymers, poly(N-isopropyl acrylamide)-b-poly(4- acryloyl morpholine)-b-poly(2-((((2-nitrobenzyl)oxy)carbonyl)amino)ethyl methacrylate) (PNIPAM-b-PNAM-b-PNBOC), which were produced through consecutive reversible addition-fragmentation chain transfer (RAFT) polymerizations. This block copolymer can self-assembly into micelles in aqueous solutions and is thermal-responsive when the solution concentration is above 2.5 g/L, and the temperature is higher than the sol-gel temperature transition (T > CGT_0,_ ~44 °C), which are the requirements for hydrogel-like structures. The PNBOC block contains a photocleavage o-nitrobenzylester moiety, which is also hydrophobic and placed in the micelle core. However, when the micelles or hydrogel are ultraviolet (UV) irradiated, the PNBOC blocks are cleaved, turning the core of the micelles into a hydrophilic core (Figure 7a). This process allows the encapsulation of hydrophobic and hydrophilic drugs in the hydrogels. The authors encapsulated hydrophobic doxorubicin (DOX) and hydrophilic gemcitabine (GCT) in the hydrogels. The author reported that the hydrogels had two different drug release patterns: (i) before UV irradiation when the hydrogel released both drugs over slower kinetics; and (ii) after UV irradiation when the drugs’ releases were fastened due to the collapse of the PNBOC (Figure 7b). Special attention should be given because the hydrogel was photoresponsive to UV light in the tests, but the gels would also be responsive to NIR irradiation, which has better tissue penetration.

Although micelles are the most well-known supramolecular structures for photoresponsive hydrogels, supramolecular nanogelators are another excellent category. Ji and colleagues [114] designed a photoresponsive coumarin-based nanogelator, which consisted of a coumarin molecule bonded to two pyridines in each extremity. When this molecule is in a water environment, it self-assembles into crystals due to noncovalent interactions like hydrogen bonds, van der Waals interactions, and π-π stacking, forming nanofibrous hydrogels. Then, bioactive molecules can be loaded into the hydrogel structure during the self-assembly process. Later, the drug release can be performed by photoirradiation of the hydrogel with UV radiation, which causes photocleavage of the coumarin-based hydrogel, disrupting the 3D-network. The coumarin-based hydrogel was loaded with methyl violet dye, a drug model, although the hydrogel was designed for anti-cancer drugs. Furthermore, drug release tests showed that the hydrogel would instantly release the dye when irradiated with UV light and sustain the drug release in a controlled manner. Altogether, the results showed that coumarin-based hydrogel could provide potential vehicles for cancer drugs once it achieves precise temporal and spatial drug release.

A very unusual method to produce hydrogel was reported by Kang et al. [115], who proposed a photoresponsive DNA-cross-linked hydrogel. The authors produced two DNA-polymer conjugates and an azo-DNA linker that consisted of complementary DNA that could be hybridized to the DNA-polymer conjugates. Then, upon DNA hybridization, the DNA-polymers conjugates can form hydrophilic tridimensional networks, conferring a hydrogel-like structure. However, because the azo-DNA linker contained azobenzene phosphoramidite, a photoisomerization moiety, the hydrogel’s sol-gel transition could be induced by light irradiation at 450 nm and be reversed (gel to sol) at 350 nm. This mechanism allowed the loading of drugs, or even nanoparticles, in the hydrogel network during the sol-gel transition and their release with the reversible gel-sol transition. The author loaded the hydrogel with DOX, trapped in the hydrogel structure, and only released it upon light irradiation. The drug release tests showed that 65% of DOX could be suddenly released up to 10 min after light irradiation.

Thermoresponsive drug delivery systems based on PTAs are very complex and multifunctional composites since most of them allow combining PTT or PDT with controlled delivery of bioactive molecules against cancer cells.

Strong and West [116] developed a drug delivery system based on gold-silica nanoshells coated with a poly(NIPAAm-co-AAm). The NIPAAm-co-AAm formulation allowed LSCT from 39 to 45 °C; in other words, the composite could be in a swollen state at physiological temperature and collapse upon heating. The gold-silica nanoshells are suitable photothermal transductor in NIR irradiation. The nanocomposite hydrogel phase was loaded with DOX, and in vitro drug release tests were performed in vials containing 1.2 × 10^9^ particles/mL, using TBS as a solution. The vials were irradiated with NIR laser for 3 min, left for 3 min when another irradiation was performed for a further 3 min (3 min on, 3 min off, 3 min on). The systems showed drug release only when the solution was irradiated with a NIR laser, suggesting a precise time resolution control. The cytotoxicity of DOX-loaded particles was compared with that from free-DOX ones using in vitro viability tests with colon carcinoma cells. Results showed that DOX-loaded particles were more cytotoxic than free-DOX ones. This effect was addressed to increased temperature caused by NIR irradiation on gold-silica nanoshells, which increased the cell membrane permeability, enhancing DOX uptake into these cells. Although the authors did not mention hyperthermia, the gold-silica nanoshells were probably performing a hyperthermic effect on the cells.

Qiu et al. [117] developed a drug delivery system based on agarose hydrogel containing black phosphorous nanosheets (BPNS) as PTA and loaded with DOX. The high photothermal conversion performed by BPNS and the thermoresponsiveness of agarose hydrogel allowed a photoresponsive hydrogel with prolonged drug release control. Drug release experiment with cycles of NIR irradiation by 10 min showed that the DOX was mostly released under a light effect. Also, the amount of drug release in each cycle was different. For example, the first cycle released almost 3-fold more DOX than the second cycle. Moreover, in vivo experiments with tumor-bearing nude mice were performed to evaluate the combination of PTT from BCNS combined with the DOX delivery. The results demonstrated that the hyperthermia effect caused by BCNS had a synergetic effect with DOX delivery, leading to much more effective treatment than only treating cancer with DOX. The same research group also studied a similar drug delivery system, but instead of BPNS, they used sodium humate (SH) [98]. The aforementioned system could also combine PTT with DOX release, but the temporal drug release control was not precise as obtained by BPNS since DOX was also significantly released when being in the off cycles of NIR irradiation.

Wu et al. [118] produced a drug delivery system based on PNIPAAm hydrogels containing poly(diketopyrrolopyrrole-alt-3,4-ethylene dioxythiophene) (PDPPEDOT) nanoparticles. PDPPEDOT is a narrow semiconductor polymer with photothermal conversion upon NIR irradiation. The DDS was loaded with DOX, and its release was performed under cycles of NIR irradiation over 20 min each 1 h up to 8 h (ON/OFF switching). An initial burst-like effect of the first NIR irradiation cycle was noted, such as that observed by Qiu et al. [117], but the next cycles showed similar DOX release, displaying high temporal and spatial drug control.

Other authors have also used organic molecules as PTA. For example, Ko et al. [119] developed a hydrogel-based on hyaluronic acid conjugated with gallic acid. The hydrogel’s gelling ability was associated with incorporating Fe^3+^ ions, which complexes with gallic acid, favoring instantaneous gelation. These complexes have a photothermal conversion and can be used to heat the hydrogel and for PTT. Although the system could be loaded with drugs, the paper only related in vivo experiments evaluating the PTT effect. The authors reported that the hydrogel was able to last more than 8 days after subcutaneous injection, and during this period, NIR irradiation could be used to perform hyperthermia (thermal ablation). The authors also reported that the hydrogel was able to treat skin cancer and solid tumors. Hydrogel injections were made in cisplatin-resistant human epidermoid carcinoma cell (KB cell) and 4T1-Luc orthotopic breast tumors in mice. Repeated NIR irradiation performed complete thermal ablation of the tumor, besides suppressing 4TI-Luc orthotopic breast tumor metastasis. Applying the hydrogel to the skin of A375 melanoma-xenografted tumor sites, followed by NIR irradiation, led to complete tumor ablation.

In another study, the photothermal conversion properties of indocyanine green (ICG) were used in thermoresponsive hydrogel based on collagen/poly(γ-glutamic acid) photoresponsive system completed with PTT [120]. The authors loaded the system with DOX and showed that the drug release could be modulated upon NIR irradiation. Moreover, the author loaded the system with MgFe_2_O_4_ nanoparticles that can be used in magnetic resonance (MR) imaging. Therefore, the authors were able to produce a theranostic platform for cancer treatment. The in vivo photothermal modulation of the MR image of the system was tested in mice and showed that the MR image could be modulated by the increase in temperature caused by ICG.

All the examples reported so far were about the combination of DDS with PTT. However, the literature has shown that it is possible to ally drug delivery systems with PDT. Xia et al. [121] produced a hydrogel formed by glycol chitosan and dibenzaldehyde-terminated telechelic poly(ethylene glycol), which was loaded with meso-tetrakis(1-methyl pyridinium-4-)porphyrin (TMPyP) that can generate ROS upon laser irradiation at 532 nm. The authors showed that TMPyP was control released by the hydrogel, and its in vitro photodynamic properties the best at an 8 μg/mL TMPyP concentration. In vivo experiments in U14 tumor-bearing mice demonstrated that the tumors treated with TMPyP loaded in the hydrogel had higher size reduction than those treated with TMPyP in PBS solution. The TMPyP emits fluorescence at 670 nm when irradiated with the laser, which could be used for imaging purposes.

In another work [122], the authors produced a multi-photoresponsive supramolecular hydrogel with dual-color fluorescence and dual-modal photodynamic action. The hydrogel’s self-assembly ability was addressed to a mixture of poly-b-cyclodextrin polymer and a hydrophobically modified dextran. On the other hand, the dual-color fluorescence and dual-modal photodynamic action were addressed to zinc phthalocyanine and a tailored nitric oxide photodonor. Therefore, this drug delivery system was able to deliver ROS and nitric oxide. Once each chemical species was released at a different photon wavelength, 520 and 420 nm, respectively, they could be delivered separately without affecting the release of one another.

### 5.4. Strengths, Limitations and Perspectives

The main strength o photoresponsive hydrogels as drug delivery systems in cancer treatment are the versatility of these systems. Regarding photoresponsive hydrogels containing photoreactive moieties, there is the advantage of producing simpler drug delivery systems, and there is no need to add extra materials to cause a photoresponse. In this sense, these drug delivery systems may be more easily designed to deliver drugs into hard-to-access sites, such as surpassing the blood-brain barrier and reaching brain tumors. On the other hand, when PTA is used in drug delivery systems, multifunctional therapeutic approaches can be performed since it may also be used in PTT or PDT. Nonetheless, in both cases—hydrogels containing photoreactive moieties or PTA—the advantage of carrying hydrophobic and/or hydrophilic drugs in the different portions of the micelles makes these drug delivery systems much more comprehensive concerning their applications in different cancer treatments, besides allowing the combination of two drugs in the same drug delivery system.

Despite the great potential of these drug delivery systems in delivering various drugs, most of the studies have studied DOX delivery, which is used as a standard drug for general cancer treatment in pharmacology-related studies. Of course, most of the researches focused on developing systems able to efficiently deliver drug with temporal and spatial resolution, rather than evaluating the efficacy of these systems in specific cancers. However, it would be interesting to focus studies on cancers using drugs designed for specialized applications. This approach could fast the approximation of photoresponsive hydrogels to clinical trials.

Undoubtedly, the main limitation of these photoresponsive hydrogels is their application in more superficial cancers once deep cancers are not reached by light incidence. Although this limitation can be overcome by employing optical fibers, it does not converge with the desire for less invasive and handle-to-use cancer treatments. Most current studies also evaluate the in vitro drug release upon light stimulus without considering the light absorbance by the skin, even though the in vivo studies focus on a subcutaneous cancer approach. In this sense, the design of in vitro drug release protocols to simulate the light absorption by the skin would lead to more reliable and consistent results.

Fortunately, some materials used as photoresponsive hydrogels are approved by FDA, such as Pluronic F127 [89]. Materials already approved by regulatory agencies are more likely to be introduced in the clinical practice faster. Considering that some PTA is already under clinical trials, like Au-based nanoparticles [22,49], probably photoresponsive hydrogels based on Pluronic F127 plus an Au-based nanoparticle may be exciting materials to explore, and move closer to clinical trials besides pre-clinical studies, which is the case of the majority of current studies.

Every year, the literature brings new hydrogels or PTA materials with improved or tailored properties for specific applications in cancer treatment. In this sense, while traditional materials like Pluronic F127 and Ag-based nanoparticles offer a quick solution to fasten photoresponsive hydrogels based on PTA to clinical practice, new materials offer unexplored opportunities, guaranteeing the development and advance of new photoresponsive hydrogels.

## 6. Concluding Remarks

This article reviewed three prominent photonic nanomedicine strategies: (i) photothermal therapy; (ii) photodynamic therapy; (iii) photoresponsive drug delivery systems. These therapies have shown promising pre-clinical results against a variety of cancers, including lung, prostate, skin, and other cancers. So far, pre-clinical in vivo results have shown promising results in tumor shrinkage upon photonic nanomedicines, but reaching deeper cancers is still a challenge to overcome. Some authors [123] have argued that reaching deep cancer might not be a problem in photonic nanomedicines since advances in fiber optics and microendoscopic technology can be designed to deliver light into the cancer site. Of course, advances in instrumentation are needed to accomplish the application of photonic nanomedicine in deeper cancers, and the development of this instrumentation may make photonic nanomedicine more comprehensive regarding its applications.

Although each of these nanomedicines has its particular type of materials and technologies, combining at least two of them has become popular since synergistic effects can be reached by combining different cancer cell death mechanisms. In general, it is a scientific consensus that some cancer treatments require a combination of different therapeutic approaches to increase the treatment efficacy. In this sense, photoresponsive hydrogels offer an exciting platform to put together different treatments in only one delivery system. Also, photoresponsive hydrogels allow the combination of photonic nanomedicines to traditional therapies like chemotherapy and immunotherapy. These kinds of multifunctional materials may be attractive if explored further. Another possibility is to ally photonic nanomedicine to traditional treatments, such as has been done with magnetic hyperthermia and other emerging nanomedicines [17,49], which have also shown a significant synergetic effect, and increase of treatment efficacy.

Therefore, photonic nanomedicine approaches might be suitable solutions for cancer treatment soon due to their effectiveness. The main advantage of these new cancer treatments is their high spatial and temporal resolution, but only clinical trials that prove their superior effectiveness over traditional cancer treatments will allow their acceptance in clinical practice. So far, both PDT and PTT are the basis of materials undergoing clinical trials, while photoresponsive hydrogels are still undergoing pre-clinical studies.

## Data Availability

No new data were created or analyzed in this study. Data sharing is not applicable to this article.
